# Roles of long non-coding RNAs in plant immunity

**DOI:** 10.1371/journal.ppat.1011340

**Published:** 2023-05-11

**Authors:** Juan Huang, Wenling Zhou, Xiaoming Zhang, Yi Li

**Affiliations:** 1 State Key Laboratory of Integrated Management of Pest Insects and Rodents, Institute of Zoology, Chinese Academy of Sciences, Beijing, China; 2 CAS Center for Excellence in Biotic Interactions, University of Chinese Academy of Sciences, Beijing, China; 3 HainanYazhou Bay Seed Lab, Sanya, China; 4 State Key Laboratory of Protein and Plant Gene Research, School of Life Sciences, Peking University, Beijing, China; Joan and Sanford I Weill Medical College of Cornell University, UNITED STATES

## Abstract

Robust plant immune systems are fine-tuned by both protein-coding genes and non-coding RNAs. Long non-coding RNAs (lncRNAs) refer to RNAs with a length of more than 200 nt and usually do not have protein-coding function and do not belong to any other well-known non-coding RNA types. The non-protein-coding, low expression, and non-conservative characteristics of lncRNAs restrict their recognition. Although studies of lncRNAs in plants are in the early stage, emerging studies have shown that plants employ lncRNAs to regulate plant immunity. Moreover, in response to stresses, numerous lncRNAs are differentially expressed, which manifests the actions of low-expressed lncRNAs and makes plant–microbe/insect interactions a convenient system to study the functions of lncRNAs. Here, we summarize the current advances in plant lncRNAs, discuss their regulatory effects in different stages of plant immunity, and highlight their roles in diverse plant–microbe/insect interactions. These insights will not only strengthen our understanding of the roles and actions of lncRNAs in plant–microbe/insect interactions but also provide novel insight into plant immune responses and a basis for further research in this field.

## Introduction

Throughout their life cycle, plants face challenges of severe environmental conditions, including diverse abiotic and biotic stresses. To overcome these challenges, plants have developed complicated immune systems to recognize stress factors and generate appropriate signal to regulate growth and development, and thus adapting to adversity [1,2]. In response to biotic stress, plants are equipped with cell-surface immune receptors and intracellular immune receptors to sense microbial signals and activate early immune responses, including calcium influx, reactive oxygen species (ROS) burst, and mitogen-activated protein kinase (MAPK) activation [3,4]. These early immune responses in turn regulate downstream transcriptional reprogramming of defense-related genes, including transcription factors and genes involved in hormone synthesis, to form late immune responses.

Long non-coding RNAs (lncRNAs) are defined as a class of endogenous single-stranded non-protein-coding transcripts with a sequence length greater than 200 nucleotides that do not belong to other well-defined non-coding RNA (ncRNA) types [5]. Advances in the past few decades have broadened our understanding of plant signal perception, activation of defense genes, and expression of resistance genes [6]. However, due to their characteristics of non-protein-coding, poor conservation among different species, stage- and cell-type specificity, and low abundance, lncRNAs failed to attract the attention of researchers in the early days. Technical innovations in genome sequencing and the development of bioinformatic tools have greatly improved our understanding of genes at the transcriptional and posttranscriptional levels [2], especially driving the discovery of ncRNAs, including lncRNAs and small RNAs (sRNAs), and furthered the exploration of their roles in regulating biological processes in animals and plants. Studies in past decades have demonstrated the important and unique roles of lncRNAs in animal growth, development, and immunity [7–9]. In comparison, studies on the function of lncRNAs in plant immunity are lagging behind. However, we should never underestimate the profound potential of lncRNAs in plant immunity. As reported by many articles, a large number of lncRNAs react to pathogen infections or insect infestations [10,11]. Therefore, biological stress could be a good system to study the actions of lncRNAs and expand our knowledge of the RNA world.

Indeed, our understanding of the roles of lncRNAs in plant immunity has improved in recent decades. LncRNAs have been shown to play critical roles in plant responses to various stresses through diverse actions. In this review, we mainly focus on the roles of lncRNAs in plant immunity, aiming to characterize the biogenesis, biological functions, and mechanisms of action of lncRNAs in different immunity stages and distinct plant–microbe/insect interactions. Overall, this review will provide novel insights into plant immunity studies and will help researchers better understand lncRNAs at multiple levels in but not limited to plant immune responses.

## Main content

### 1. Biogenesis and modes of action of plant lncRNAs

LncRNAs are ubiquitously present in almost all forms of life ranging among animals, plants, fungi, and prokaryotes, and even including viruses. With the development of genome sequencing and bioinformatic analysis tools, enormous lncRNA candidates have been identified in different plants, including *Arabidopsis* [12–14], rice [15–21], maize [22–24], cotton [25,26], Medicago [27], etc. [28]. These lncRNA candidates can be found in many plant databases, including the general plant databases, such as TAIR and Araport, and specific non-coding RNA databases, such as PLncDB, Green Non-coding Database (GREENC), NONCODE, CANTATAdb, PNRD, and PlantNATsDB [29].

**Fig 1:**
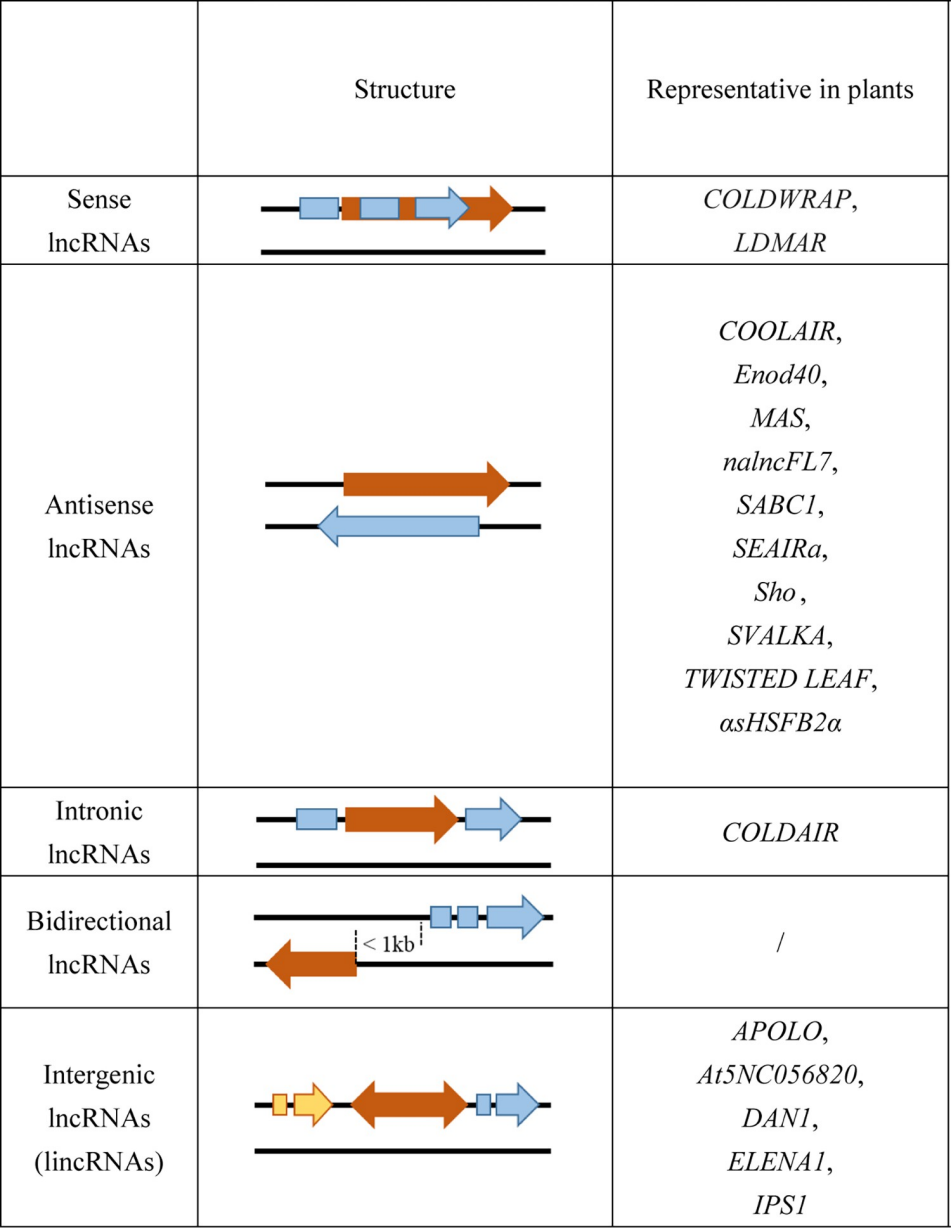
Classification of lncRNAs based on their genomic location to protein-coding genes. LncRNAs were classified into 5 categories. Representatives of each category were listed in the figure, such as COLDWRAP [44] and LDMAR [74] in sense lncRNAs, COOLAIR [85], Enod40 [52], MAS [158], nalncFL7 [142], SABC1 [45], SEAIRa [159], Sho [83], SVALKA [86], TWISTED LEAF [147], and αsHSFB2α [62] in antisense lncRNAs, COLDAIR [43] in intronic lncRNAs, and APOLO [157], At5NC056820 [12], DAN1 [84], ELENA1 [148], and IPS1 [56] in intergenic lncRNAs. Blue square and arrow represent Exons of gene A; yellow square and arrow represent Exons of gene B; brown square represents lncRNA. The direction of the gene A, gene B, and lncRNA are showed by the direction of the arrow. lncRNA, long non-coding RNA.

As the most abundant class of ncRNAs, lncRNAs are key regulators of gene expression in various biological processes [30]. According to the positional relationship between an lncRNA and its neighboring protein-coding genes on chromosomes, lncRNAs can be divided into 5 groups (Fig 1): (1) sense lncRNA: located on the same strand as its associated protein-coding gene, and partially or completely, overlapping with the coding region; (2) antisense lncRNA: located on the opposite strand of its associated gene, and partially or completely, overlapping with the coding region; (3) intronic lncRNAs: located within an intron of the associated protein-coding gene; (4) bidirectional lncRNA: located on the opposite strand of the associated protein-coding gene at a distance less than 1 kb from the promoter of the protein-coding gene; and (5) long intergenic non-coding RNA (lincRNA): transcribed from the intergenic region between 2 protein-coding genes [31,32]. Most of well-characterized lncRNAs in plants are antisense lncRNAs and lincRNAs, while rare bidirectional lncRNAs have been characterized in plants (Fig 1).

Generally, the biogenesis of lncRNAs is similar to that of mRNAs. LncRNAs are usually transcribed by RNA polymerase (Pol) II from intergenic, exonic, or the distal protein-coding regions of the genome [5,33,34]. After transcription, they undergo 5′-end capping, 3′-end polyadenylation, and sometimes alternative splicing [35]. Interestingly, some non-polyadenylated lncRNAs have been identified and appear to be more specific to the stress response [10]. In addition to Pol II, Pol III, Pol IV, and Pol V can transcribe plant lncRNAs [36,37]. Pol III usually produces relatively short, high-quantity and stable RNAs, such as 5S rRNA and tRNA. Interestingly, lncRNAs *AtR8* and *AtR18* were efficiently transcribed by Pol III *in vitro* in tobacco nuclear extracts, with *AtR8* being shown to be a functional lncRNA conserved in *Brassicaceae* and acting in responses to different stress treatments [38]. The lncRNAs transcribed by Pol IV and Pol V have structural differences compared to those transcribed by Pol II, such as lacking a poly (A) tails [36,39]. LncRNAs that transcribed by Pol IV usually serve as RDR2 templates for the synthesis of 24-nt sRNAs, whereas lncRNAs produced by Pol V function as a scaffolds to recruit 24-nt sRNAs to their complementary target loci in the genome [40,41]. Compared with lncRNAs transcribed by Pol II, lncRNAs transcribed by RNA Pol IV or Pol V are poorly characterized. Their low expression and high instability make them more difficult to identify and characterize.

LncRNAs modulate the expression of their target genes in *cis*, in *trans*, or through other actions. *Cis-*acting lncRNAs usually regulate the transcription of genes in close genomic proximity by recruiting or displacing transcription factors at the promoters of neighboring genes [29]. The three-dimensional organization of genomes plays key roles in the transcriptional regulation of genes. Some *cis-*acting lncRNAs interact with chromatin remodeling complexes and modulate the three-dimensional organization of genomes, such as forming chromatin loops with target genes, to affect histone modifications and transcriptions of target genes [42–46]. *Trans-*acting lncRNAs, however, usually target genes far from the site of the primary locus of transcription, acting as a scaffold of protein complexes to recruit transcriptional or chromatin-modifying factors, or as a platform to assemble protein complexes [47–52]. In addition, lncRNAs can interact with proteins to modulate their activity, stability, or subcellular localization [52,53]. Moreover, lncRNAs could act as precursors of some sRNAs to modulate the expression of mature sRNAs or functions as decoys of sRNA to interfere RNA silencing to regulate gene expression [54–57]. For lncRNAs that exhibit a coordinated expression profile with their neighboring genes (*cis-*acting lncRNAs), it is essential to distinguish the function of lncRNAs from that of their neighboring genes. Therefore, generating proper lncRNA mutants without directly affecting the function of neighboring genes is most important. For *trans-*acting lncRNAs, it is essential to find the primary targets.

### 2. Regulatory roles of lncRNAs in plant immunity

**Fig 2:**
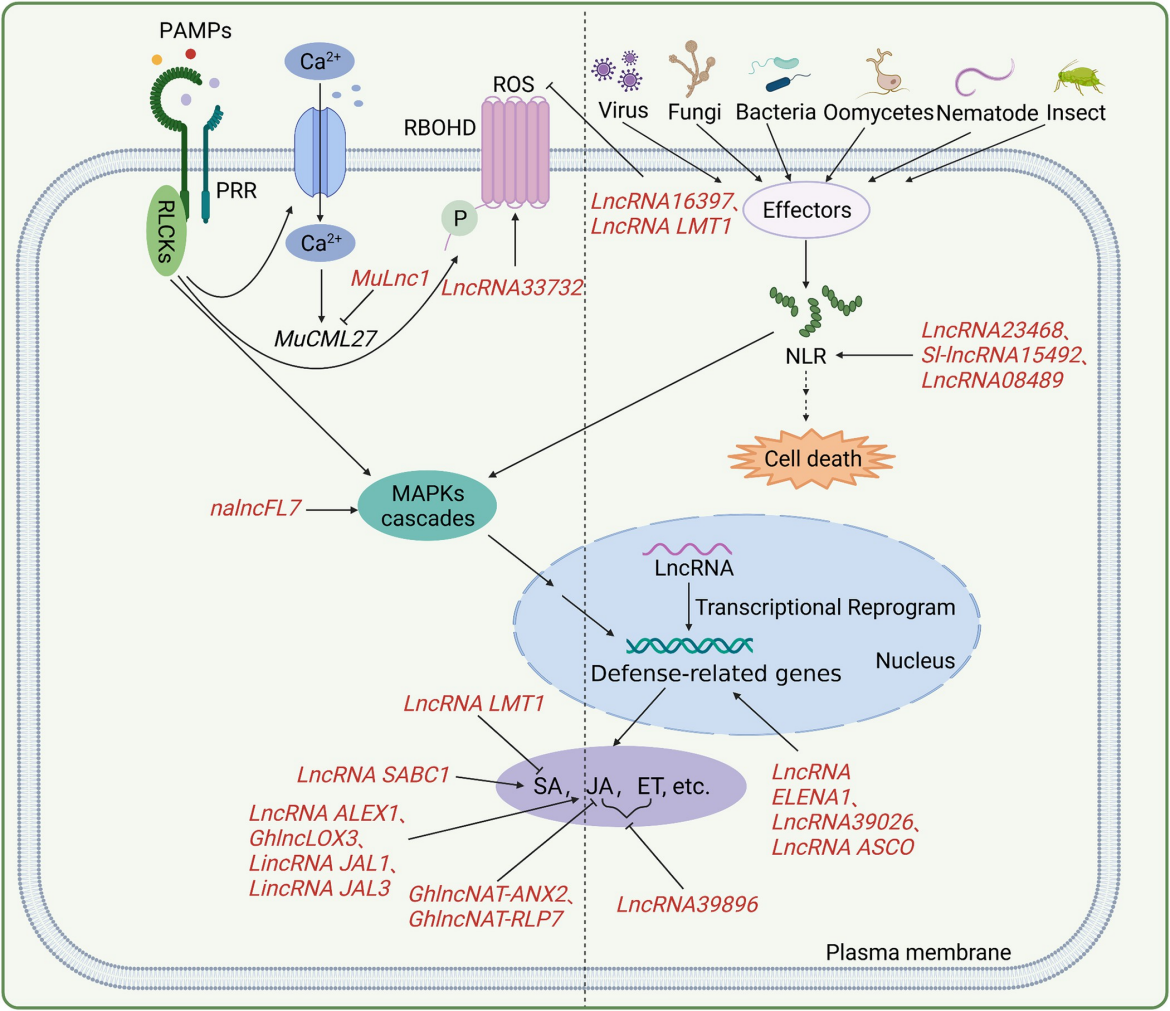
LncRNAs in plant immunity. Pathogens and insects activate plant PRRs or produce effectors to activate NLRs and further trigger different signaling events and immune defense mechanisms. Pathogen infection changes the expression of lncRNAs, and the differentially expressed lncRNAs regulate various aspects of plant immunity, including ROS accumulation, calcium influx, MAPK activation, hormone pathway activity, and defense-related gene expression. Created with Biorender.com. lncRNA, long non-coding RNA; MAPK, mitogen-activated protein kinase; PRR, pattern recognition receptor; NLR, nucleotide-binding domain, leucine-rich–repeat-containing receptors; ROS, reactive oxygen species.

The enormous lncRNAs in plants form regulatory networks with protein-coding genes, and/or other non-coding RNAs to mediate growth, development, stress responses, and other biological processes. Coupled with their important roles, the expression of lncRNAs is stage- and cell-type specific and tightly regulated in response to abiotic or biotic stimuli, which subsequently facilitates plants to cope with these stimuli. Abiotic stimuli that result in the differential expression of lncRNAs have been reported in *Arabidopsis* [10,38,58–67], wheat [68], barley [69,70], rice [71–74], maize [75–80], etc. [81–84]. For example, in response to cold, the lncRNAs *COLDWARP*, *COOLAIR*, and *COLDAIR* are induced and regulate vernalization by transcriptional silencing of *FLOWERING LOCUS C* (*FLC*) [43,44,85]. The level of the lncRNA *SVALKA* was found to gradually increase during the early responses to cold temperatures and to promote cold acclimation by fine-tuning the expression of Crepeat/dehydration-responsive element Binding Factor 1 (*CBF1*) [86]. Similarly, the expression patterns of lncRNAs also react in a genome-wide manner to biotic stimuli, which in turn modulates the resistance of plants to different pathogens [87–91]. However, due to the specific function and action of each lncRNA, the mechanism by which lncRNAs regulate plant immunity remains scant. Here, we summarize the current knowledge about the roles of lncRNAs in different stages of plant immunity (Fig 2).

### 2.1. Roles of lncRNAs in immune perception processes

To properly activate immune defense, plants have developed cell-surface receptors and intracellular receptors to perceive signals from pathogens. Generally, plant cell-surface pattern recognition receptors (PRRs) perceive immunogenic signals from microbes/insects or host-derived molecular patterns, whereas canonical plant intracellular nucleotide-binding domain, leucine-rich–repeat-containing receptors (NLRs) sense the presence of a pathogen effector by directly interacting with effectors that are secreted into plant cells, or recognize changes of guard host proteins, the replication of viruses/pathogens, integrated diverse cues, etc. [92–97]. However, there are a few cell-surface receptors that detect highly specific effector signatures, such as tomato Cf9, which recognizes AvrCf9 [98,99], while a few NLRs recognize other signatures, in addition to pathogen effectors (e.g., a canonical NLR, N, recognizes the replicase protein of Tobacco mosaic virus, p50) [100].

Many immunogenic signals recognized by PRRs have been identified, among which the most commonly studied are bacterial flagellin, bacterial elongation factor-Tu, and fungal chitin [92–94,101]. In addition, the damage to plant tissues, particularly the plant cell wall, caused by enzymes or toxins of pathogens, as well as the immunogenic peptides produced by plants, is recognized by PRRs [102–105]. Studies have shown that the expression levels of some lncRNAs are significantly altered after treatment with immunogenic signals (Fig 2). For example, in *Arabidopsis*, the accumulation of the lncRNA *At5NC056820* was found to be increased by 22-fold after the treatment with elf18 (Elongation factor-Tu, EF-Tu) [12]. Likewise, in response to the treatment with flg22 or *Pseudomonas fluorescens* 55, many lncRNAs in tomato were shown to be up- or down-regulated, and the number of differentially expressed lncRNAs was dramatically increased at 6 h post inoculation [106].

During the coevolution of plants and pathogens, pathogens have evolved diverse effectors to facilitate pathogens to overcome the basic immune response of plants [107,108]. Plant NLRs recognize effectors either through direct physical interaction or sensing of host protein modifications caused by effectors and subsequently activate immune responses [102,109–113]. In healthy plants, *NLRs* are suppressed to balance plant growth and immunity [114]. Conserved regions of *NLR* genes are widely targeted by microRNAs (miRNAs) and phasiRNAs, especially 22-nt microRNAs, to repress plant immunity under normal conditions [115–121]. Upon pathogen infection, the accumulation of these 22-nt microRNAs decreases, which releases the accumulation of miRNA-targeted *NLR* genes and thus increases plant immunity [115,120]. LncRNAs have also been shown to be differentially expressed corresponding to the activation of NLRs (Fig 2). Genomic analysis revealed 145 up- and 118 down-regulated lncRNAs in response to *AvrPto* and *AvrPtoB*, 2 well-studied *Pseudomonas syringae pv*. *tomato* (*Pst*) DC3000 effectors that could interfere with PRR signaling [106]. Some of these lncRNAs modulate the expression of *NLR* genes through their interactions with miRNAs that target *NLR* genes. For example, miR482 targets the coiled-coil domains of the N terminal of *NLR* genes in *Solanum* species [115]. Tomato *lncRNA23468*, which contains conserved endogenous target mimic sites for miR482b, was shown to suppress miR482b expression to up-regulate the expression of *NLR*s, and thereby enhancing tomato resistance to *Phytophthora infestans* [122]. On the other hand, the overexpression of tomato *lncRNA15492* and *lncRNA08489* resulted in increased the expression of *NLRs*, corresponding with decreased expression of miR482a and miR482e-3p, respectively, and subsequently enhanced plant resistance to *P*. *infestans* [123,124]. LncRNAs appear to regulate plant immunity by acting as decoys of sRNAs or sRNA precursors to mediate the expression of *NBS-LRR* resistance genes.

### 2.2. Roles of lncRNAs in immune responses

Immune responses triggered by cell-surface immunogenic signals and intracellular pathogen effectors have obvious differences in their mechanisms of action, but they also have mutual relations, and have developed into an interconnected mode of action in the coevolution of plants and pathogens [125,126]. The activation of PRRs phosphorylates immediate downstream receptor-like cytoplasmic kinases (RLCKs) and leads to a subsequent series of downstream signaling events, including ROS accumulation, calcium influx, MAPK phosphorylation cascades, defense gene expression, stomata closure, callose deposition, and biosynthesis of defense hormones [4,127–130]. The physiological responses mediated by NLRs overlap with those induced by PRRs, such as increased ROS production, activation of MAP kinases, but are delayed, stronger and prolonged, which usually leads to programmed cell death, known as the hypersensitive response (HR) [131]. These immune responses are fine-tuned not only by protein-coding genes but also by lncRNAs (Fig 2).

#### 2.2.1. Roles of lncRNAs in the accumulation of ROS

Studies have found that lncRNAs alter the accumulation of ROS by regulating the expression of genes in close genomic proximity [132,133]. Tomato *lncRNA16397* reduces ROS accumulation, alleviates cell membrane injury, and subsequently enhances plant resistance to *P*. *infestans*, probably by inducing the expression of its neighboring gene *SlGRX* [132]. Meanwhile, tomato *lncRNA33732* was reported to induce the expression of respiratory burst oxidase (RBOH) to increase the accumulation of H_2_O_2_ during early defense against *P*. *infestans* attack [133].

#### 2.2.2. Roles of lncRNAs in calcium influx

Transient and rapid calcium influx upon infection is important for early cellular responses in plant immunity and essential for triggering downstream signaling [134]. Currently, no lncRNA has been identified to directly regulate calcium influx, but some lncRNAs have been found to act downstream of calcium influx. *MuLnc1* in mulberry forms a mulmiR3954-*MuLnc1*-siRNAs-mRNAs network to enhance resistance to *Botrytis cinerea* and *Pst* DC3000 [55]. When cleavaged by mulmiR3954, *MuLnc1* was found to produces si161579, a siRNA that cleavages the transcript of the calmodulin-like protein gene *CML27*. *CML27* belongs to the CML family whose members are important Ca^2+^ sensors. Therefore, the lncRNA *MuLnc1* may act downstream of calcium influx via *CML27*. ROS and calcium influx also contributes to the down-regulation of the lncRNA salicylic acid biogenesis controller 1 (*SABC1*) upon pathogen treatment [45].

#### 2.2.3. Roles of lncRNAs in the activation of MAPK cascades

The activation of MAPK cascades is a major early signaling event downstream of PAMP perception and response for the transduction of extracellular stimuli into intracellular responses [135,136]. The activation of MAPK cascades triggers multiple defense responses, including regulating the transcription of defense-related genes, immune signaling proteins, and biosynthetic enzymes of defense hormones, ROS generation, cell wall strengthening, and HR cell death [137]. The activity of MAPK is regulated by the dephosphorylation of protein serine/threonine phosphatases (PSPs) [138]. Effectors employed by pathogens could suppress the activation of MAPK to attenuate resistance [139–141]. Recently, a *nalncFL7*-*FL7*-HAI1-MAPK3/6 cascade was reported to regulate MAPK cascade immunity responses [142]. The *cis-*natural antisense lncRNA of *FL7* (*nalncFL7*) is protected by BPL3, a conserved negative regulator of plant immunity, and suppresses the accumulation of *FL7* transcripts. In response to pathogens, the transcript levels of *BPL3* decrease, resulting in the degradation of *nalncFL7* and thus releasing its suppression on *FL7*. *FL7* interacts with HIGHLY ABA-INDUCED PP2C1 (HAI1), a kind of PSPs, and inhibits the phosphatase activity of HAI1. By decreasing the phosphatase activity of HAI1, *FL7* increases the phosphorylation levels of MPK3 and MPK6, which enhances immunity responses.

#### 2.2.4. Roles of lncRNAs in altering the defense-related gene expression

PRRs and NLRs triggered immune responses involve the activation of a series of overlapping downstream defense responses [131]. Many of these reactions transmit signals from the cell membrane to the nucleus, where these signals modulate the transcriptional level of some defense related genes, pathogenesis related (*PR*), lipoxygenase (*LOX*), phenylalanine ammonia-lyase (*PAL*), catalase (*CAT*), *GDSL* lipase, antimicrobial peptides (A*MP*s), etc. [143–146]. Considering the low accumulation of lncRNAs, transcriptional reprogramming of genes is one of the profound functions of lncRNAs to manifest the actions of lncRNAs. LncRNAs target or interact with transcription factors, splicing factors, epigenetic regulators, and some other key proteins to modulate their activities and regulate the expression of genes in downstream signaling pathways.

Due to the profound roles of transcription factors in transcriptional reprogramming and hormone activation, lncRNAs targeting nearby transcription factors to efficiently exert their actions have been well characterized, such as the lncRNAs *COOLAIR*, *COLDAIR*, and *COLDRAP* to *FLC* in *Arabidopsis* [43], and the lncRNAs *TWISTED LEAF* to R2R3-MYB in rice [147]. A recent study identified 15 defense-related transcription factors in *Arabidopsis* that may be targeted by adjacent lncRNAs [45]. Among these lncRNAs, the lncRNA *SABC1* represses the transcriptional level of its neighboring gene *NAC3*, a NAC transcription factor, to repress plant immunity in healthy plants. Upon pathogen infection, calcium influx and ROS burst decrease the accumulation of *SABC1*, release the expression of *NAC3* to activate transcriptional reprogramming and hormone activation, thus tilting the balance from plant growth to plant immunity. In addition to modulating the expression of adjacent genes, lncRNAs can act *in trans* to regulate the activity of transcription factors. In addition to modulating adjacent genes, the *Arabidopsis* lncRNA *ELF18-INDUCED LONG-NONCODING RNA1* (*ELENA1*) was shown to increase plant resistance against *Pst* DC3000 by directly interacting with the mediator subunit 19a (MED19a), a positive regulator, to enrich MED19a on the PR1 promoter, then inducing PR1 expression [148,149]. Furthermore, *ELENA1* also interacts with FIB2 (MED36a), a transcriptional repressor, to release MED19 from the FIB2/MED19a complex, and the dissociation of FIB2 from MED19 results in the full activation of PR1 expression by MED19 [148]. Moreover, a genome-wide analysis of lncRNA and miRNA networks in tomatoes upon *P*. *infestans* infection identified lncRNAs that were predicted to decoy miRNAs and modulate the transcription of target genes, including transcription factors [150]. *LncRNA42705*/*lncRNA08711*, *lncRNA39896*, and *lncRNA11265*/*lncRNA15816* were predicted to decoy miR159, miR166b, and miR164a-5p, respectively, and to modulate the transcriptional level of MYB, HD-Zip, and NAC transcription factors, respectively. These transcription factors further regulated the expression of defense-related genes and altered the plant response to pathogens.

In addition to targeting or regulating transcription factors, lncRNAs interact with splicing components to fine-tune the plant transcriptional response to pathogens. In response to flagellin, the *Arabidopsis* lncRNA *ALTERNATIVE SPLICING COMPETITOR* (*ASCO*) interacts with the spliceosome-core components PRP8a and SmD1b, alters SmD1b/PRP8a-dependent transcriptome diversity, differentially alternatively splices flg22-response regulatory genes, and subsequently attenuates root growth sensitivity to flg22 [151]. The lncRNA *ASCO* was also found to hijack the alternative splicing (AS) regulators NUCLEAR SPECKLE RNA-BINDING PROTEINS (NSRs) to modulate the AS of NSR targets and alter the plant response to auxin [52]. Interestingly, *ASCO* presented different regulatory mechanisms in response to flagellin, a peptide released by bacteria and acting as a triggering PAMP, and auxin, a hormone that balances plant growth and immunity. Further study suggests that other lncRNAs than *ASCO* may also interact with NSRs to modulate AS [152]. The participation of lncRNAs in plant development and immunity may be far more complicated than current model.

LncRNAs can also regulate gene transcription by interacting with chromatin regulatory proteins, including CURLY LEAF (CLF), LIKE HETEROCHROMATIC PROTEIN 1 (LHP1), etc., to regulate the chromatin topology on a genome-wide scale. The modified chromatin topology recruits regulatory protein/lncRNA complexes to specific sites on DNA and performs chromatin modification [153–155]. The repression of lncRNA *COLDAIR*, *COLDWRAP*, *COOLAIR*, and *AG-incRNA4* on *FLC* was performed by lncRNAs interacting with CLF, a key component of polycomb repressive complex 2 (PRC2), catalyzing histone H3 lysine 27 trimethylation (H3K27me3) of *FLC*, and repressing its transcription [43,44,85]. Among them, COLDAIR and COLDWRAP cooperatively formed chromatin loops between the promoter and the 3′ end of the first intron of *FLC* to maintain the polycomb-mediated silencing of *FLC*. The lncRNA *AUXIN REGULATED PROMOTER LOOP* (*APOLO*) associates with LIKE HETEROCHROMATIC PROTEIN 1 (LHP1), the key component of PRC1, forming a chromatin loop to encompass the intergenic region between the APOLO loci and its neighboring gene PINOID, and thus regulating the expression of PINOID [156,157]. The lncRNA *SABC1*, which is down-regulated in response to *Pst* (*avrRpt2*) inoculation and *Turnip mosaic virus* (TuMV) infection, represses the transcription of *NAC3* by associating with CLF and recruiting CLF/PRC2 complexes to increase the H3K27me3 of *NAC3*, which subsequently decreases the association of Pol II to *NAC3* promoter [45]. Although *COLDAIR/COLDWRAP*, *APOLO*, and *SABAC1* all form a repressive chromatin loop to associate with target genes, lncRNAs are required for the formation of the chromatin loop of *COLDAIR/COLDWRAP-FLC* and *APOLO-PINOID*, but this is not the case for the *SABC1-NAC3* loop. The chromatin loops of *COLDAIR/COLDWRAP-FLC* and *APOLO-PINOID* are unstable during vernation and auxin treatment, respectively, while the loop at the *SABC1-NAC3* locus is stable upon pathogen infection [42,43,156,157]. The general roles of lncRNAs in the formation of chromatin loops need to be further determined. In addition, other chromatin regulatory proteins were revealed to interact with lncRNAs and induce chromatin modification of target genes. The lncRNA *MAS* interacts with WDR5a, a core component of COMPASS-like complexes, and recruits WDR5a to *MAF4* to enhance H3K4me3, thus activating *MAF4* [158], while the intragenic lncRNA *SEAIRa* interacts with PUB25/26 and RUB1 and induces H3K27me3 and H2A monoubiquitination (H2Aub) deposition on its neighboring target SE to cause transcriptional and epigenetic repression of SE [159].

#### 2.2.5. Roles of lncRNAs in regulating defense-related hormones and hormone pathways

Plant hormones, including salicylic acid (SA), jasmonic acid (JA), ethylene (ET), gibberellin (GA), and abscisic acid (ABA), regulate plant defense against pathogens, among which SA and JA are major defense hormones. SA plays essential roles in resistance against biotrophic and hemi-biotrophic pathogens and some phloem-feeding herbivores, whereas JA is critical in defense against necrotrophic pathogens, some phloem-feeding herbivores, and chewing herbivores [160,161]. SA and JA often function antagonistically [162]. In basal resistance, SA blocks JA production and JA-mediated gene activities. However, in NLR-induced immunity, the initial activation of JA-responsive genes is dependent on SA and SA receptors. The interplay between SA and JA allows the plant to generate defense against different pathogens [163,164].

In *Arabidopsis*, the pathogen-induced production of SA requires 3 proteins: isochorismate synthase 1 (ICS1), which converts chorismate into isochorismate in plastids; enhanced disease susceptibility 5 (EDS5), which transports isochorismate from plastids to the cytosol; and AVRPPHB susceptible 3 (PBS3), which conjugates isochorismate with glutamate to form isochorismate-9-glutamate. The degradation of isochorismate-9-glutamate spontaneously produces SA and 2-hydroxy-acryloyl-*N*-glutamate [165]. Pathogen-induced ICS1 expression and SA biosynthesis are tightly regulated by positive and negative transcription factors [166,167]. The lncRNA *SABC1* was found to regulate the biosynthesis of SA to modulate plant immunity [45,167]. Upon pathogen infection, the activation of calcium influx and ROS burst decrease *SABC1* accumulation and subsequently activate *NAC3*. The activation of *NAC3* then promotes the biosynthesis of SA by binding to the promoter of *ICS1* [45]. The profound roles of SA in the induction of defense-related genes and amplification of immune signaling allow *SABC1* to mediate the balance between plant defense and growth [45,167]. Furthermore, the lncRNA *AtR8*, which can be induced by low-level SA, was also found to participate in SA response-related defense upon *P*. *syringae* infection [168].

JA, a vital plant hormone essential for plant defense responses and developmental processes, exhibits diverse responses to different biotic stresses [169,170]. The JA-mediated defense system enhances host defense against insect herbivores and necrotrophic fungi, such as *Alternaria brassicicola*, *B*. *cinerea*, *Plectosphaerella cucumerina*, and *Fusarium oxysporum* [171,172]. LncRNAs have been reported to participate in these regulatory processes. Invasion by *V*. *dahliae* was shown to increase the expression of the lncRNA *GhlncLOX3* and subsequently improve plant resistance, probably through the repressive effect of *GhlncLOX3* on the transcription level of *GhLOX3* (lipoxygenase 3, a JA pathway gene) and lipoxygenase 2 (LOX2), and JA content [173]. JA mediates plant defense through the regulation of CORONATINE INSENSITIVE1 (COI1)-JASMONATE-ZIM-DOMAIN (JAZ)-transcription factors signaling cascades [174,175]. Many transcriptional activators and repressors in the JA response pathway have been identified, including *MYC2*, *MYC3*, *MYC4*, basic-helix-loop-helix (bHLH) *3*, *bHLH13*, *bHLH14*, and *bHLH17/JAM1* [176–179]. An lncRNA, *An Leaf Expressed* and *Xoo- induced lncRNA 1* (*ALEX1*), was identified to be specially induced by X*anthomonas oryzae pv*. (*Xoo*) infection in rice [180]. The expression of *ALEX1* significantly up-regulates JA-related genes such as *JAZ8*, *MYC2*, *PR1a*, *PR1b*, *PR10a*, and *RSOsPR10*, and increases the endogenous levels of JA, conferring broad-spectrum resistance to bacterial pathogens. Correspondingly, some other enzymes and transcription factors in the JA biosynthetic signaling pathway are hijacked by pathogens to attenuate plant immunity [181,182]. Two cotton lncRNAs, *GhlncNAT-ANX2* and *GhlncNAT-RLP7*, have been found to be induced by the infection with *Verticillium dahliae* or *B*. *cinerea*, repress the expression of 2 JA pathway genes, lipoxygenase 1 (LOX1) and lipoxygenase 2 (LOX2), and further attenuate plant resistance against fungi [183]. *LncRNA39896* in tomatoes, which is induced by *P*. *infestans* infection, act as endogenous target mimic of miR166b and negatively regulates tomato resistance through the *lncRNA39896*–miR166b–HDZs module [184]. In *lncRNA39896*-knockout mutant, miR166b activity is increased, resulting in increased cleavage of *SlHDZ34* and *SlHDZ45*, and increased JA and ET contents, which was not favorable for *P*. *infestans* infection. However, the molecular mechanism underlying the regulation is still unclear.

### 3. Roles of lncRNAs in various plant–microbe/insect interactions

**Fig 3:**
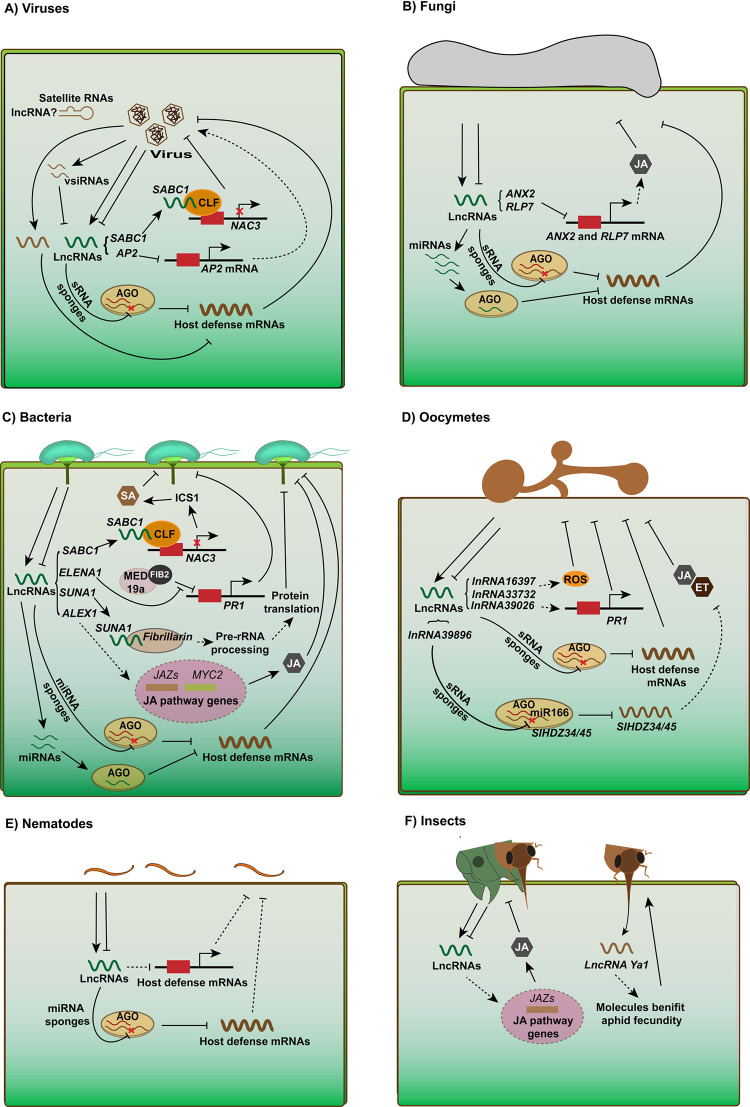
Roles of lncRNAs during plant–pathogen/insect interactions. Attack by pathogens/insects significantly changes the expression of lncRNAs, and these lncRNAs function in plant immunity through different mechanisms. (A) In response to viral infection, lncRNAs can act as sponges of sRNAs to regulate the expression of host defense mRNAs and further mediate plant immunity. In addition, the lncRNA *SABC1*, which represses the transcription of its neighboring gene, *NAC3*, by interacting with CLF and increasing the H3K27me3 of *NAC3*, is down-regulated during TuMV infection and plays a negative role in plant resistance to virus. The lncRNA *AP2*, which is up-regulated by TCV, promotes the infection of TCV, probably by regulating its neighboring gene, *AP2*. As a counter strategy, viruses can produce vsiRNAs to silence host lncRNAs, to attenuate host immunity. Moreover, some non-coding satellite RNAs are considered to be function as lncRNAs. (B) In response to fungal infection, lncRNAs can regulate plant immunity by acting as precursors of miRNAs or sponges of miRNAs to indirectly inhibit the cleavage of miRNA target genes. In addition, the lncRNAs *ANX2* and *RLP7* in cotton decrease the expression of their neighboring genes, regulate the JA response by affecting the JA pathway genes, *LOX1* and *LOX*, and promote the infection of *V*. *dahliae* and *B*. *cinerea*. (C) During plant–bacteria interactions, lncRNAs regulate the expression of defense mRNAs to mediate plant defense by acting as precursors of miRNAs or sponges of miRNAs. Moreover, the expression of *SABC1* is suppressed in response to *Pst* DC3000 infection, and its suppression triggers the transcription of *NAC3* and biosynthesis of SA, thus activating plant resistance. *ELENA1* enhances the resistance of *Arabidopsis* to *Pst* DC3000 by interacting with the mediator subunit 19a and FIB2 to promote the gene expression of PR1, while the lncRNA *SUNA1* promotes plant defense against *Pst* DC3000 by interacting with fibrillarin to enhance the pre-rRNA processing and translational efficiency of some defense genes. In addition, the lncRNA *ALEX1* enhances rice resistance to *Xoo* by up-regulating the endogenous levels of JA and expression of JA-responsive genes. (D) LncRNAs mediate plant defense against oocymetes by affecting ROS accumulation, changing the expression of PR genes, or acting as decoys of miRNAs. Many lncRNAs serve as positive regulators of plant immunity in response to oocymetes by acting as decoys of miRNAs, while lncRNA39896 negatively regulates plant defense by inhibiting miR166 activity. (E) In response to nematodes, lncRNAs interact with their corresponding miRNAs, exerting miRNA-related regulatory effects, or may regulate host defense mRNAs through other mechanisms. (F) In response to insect attack, lncRNAs are involved in the regulation of JA accumulation, probably by mediating the gene expression of JAZ genes. On the other hand, the aphid transcripts *Yas* serves as an lncRNA when being translocated into plants and promotes the fecundity of aphids. RNAs produced by pathogens/insects are shown in brown. JA, jasmonic acid; lncRNA, long non-coding RNA; ROS, reactive oxygen species; sRNA, small RNA.

Plants have evolved sets of defense mechanisms to effectively mitigate different diseases. We next summarized the roles of lncRNAs in various plant–microbe/insect interactions, including viruses, fungi, bacteria, oomycetes, nematodes, and insects (Fig 3). The well-studied lncRNAs that are categorized into different plant–microbe/insect interactions are listed in Table 1.

**Table 1:** List of lncRNAs associated with plant immunity.

Category	LncRNA	LncRNA accumulation alteration upon stress	Host	Stress association	Target genes	Function/Mechanism	Reference (PMID)
Virus	*LINC-AP2*	up	*Arabidopsis*	*Turnip crinkle virus*	*APETALA2*	Promotes *TCV* infection in *Arabidopsis* probably by down-regulating the expression of *AP2* gene.	[187]
	*LncRNA LMT1*	up	Tobacco	*Citrus tristeza virus*	*AOX-1a*	Produced by *CRV*, stimulates host *AOX-1a* expression, suppresses SA and ROS accumulations, and weakens immunity.	[195]
	LncRNA *S-slylnc0957*	up	Tomato	*Tomato yellow leaf curl virus*	?	Negatively regulates plant resistance to TYLCV.	[185]
	*slylnc0049*, *slylnc 0761*	up	Tomato	*Tomato yellow leaf curl virus*	?	Promote the infection of TYLCV.	[88]
	*slylnc0195*	up	Tomato	*Tomato yellow leaf curl virus*	class III HD-Zip genes	Act as miR166 sponges to inhibit the TYLCV infection.	[88]
	*slylnc1077*	up	Tomato	*Tomato yellow leaf curl virus*	*Solyc09g082060*.*2*.*1*	Act as miR399 sponge to regulate the TYLCV infection.	[88]
Fungus	*GhlncNAT-ANX2*, *GhlncNAT-RLP7*	up	Cotton	*Verticillium dahlia*/*Botrytis cinerea*	*ANX2*, *RLP7*	Attenuate cotton defense against cotton fungal disease, possibly by decreasing the expression of their neighboring genes *ANX2* and *RLP7*, respectively. Involved in decreasing the expression of 2 JA pathways genes, *LOX1* and *LOX2*.	[183]
	*GhlncLOX3*	up	Cotton	*Verticillium dahlia*	*GhLOX3*	Improves plant resistance to fungi by increase the expression of *GhLOX3* gene and JA content.	[173]
Bacterium	*LncRNA ALEX1*	up	Rice	*Xanthomonas oryzae pv*. *Oryzae*	*JAZ8*, *MYC2*, *PR1a*, *etc*.	Up-regulates JA and JA-responsive genes and enhance rice resistance to bacterial blight.	[180]
	*LncRNA ELENA1*	up	*Arabidopsis*	*Pst* DC3000	*PR1*, *PR2*, *BG3*, *CYP82C2*, *etc*.	Interacts with MED19a and FIB2 to increase *PR1* transcription and plant resistance.	[148]
	*LncRNA SABC1*	down	*Arabidopsis*	*Pst* D3000*/Turnip mosaic virus*	*NAC3*	Represses plant immunity to bacteria and virus by inhibiting *NAC3* transcription and attenuating SA biosynthesis.	[45]
	*LncRNA SUNA1*	up	*Arabidopsis*	*Pst* DC3000	*EDR1*, *SARD1*, *PAD4*, *EDS1*, *EDR4* and *ACD6*	Induced by *Pst* DC3000 through SA and increases plant resistance by regulating pre-rRNA processing and translational efficiency of defense genes.	[209]
Oomycete	*LncRNA08489*	up	Tomato	*Phytophthora infestans*	*NBS-LRR*	Enhances tomato resistance through decoying miR482e-3p and modulating the accumulation of *NBS-LRR*.	[124]
	*LncRNA16397*	up	Tomato	*Phytophthora infestans*	*SlGRX21*	Enhances resistance to *P*. *infestans* by inducing SlGRX expression, reducing ROS accumulation, and alleviating cell membrane injury.	[132]
	*LncRNA23468*	up	Tomato	*Phytophthora infestans*	*NBS-LRRs*	Increases *NBS-LRRs* expression by decoying miR482b and enhances tomato resistance to *P*. *infestans*.	[122]
	*LncRNA33732*	up	Tomato	*Phytophthora infestans*	?	Enhances tomato resistance to *P*. *infestans* by inducing the expression of respiratory burst oxidase and increasing the accumulation of H_2_O_2_.	[133]
	*LncRNA39026*	up	Tomato	*Phytophthora infestans*	*SlPR1*, *SlPR2*, *SlPR3*, *SlPR5*	Enhances tomato resistance to *P*. *infestans* by decoying miR168a and inducing *PR* gene expression.	[189]
	*LncRNA39896*	up	Tomato	*Phytophthora infestans*	*SlHDZ34 SlHDZ45*	Suppresses tomato resistance to oocymete by acting as endogenous target mimic of miR166b to increase transcript level of *SlHDZ34* and *SlHDZ45*, and performing JA and ET regulation.	[184]
	*LncRNA40787*	up	Tomato	*Phytophthora infestans*	*LCR*	Enhancing tomato resistance by decoying miR394 and decreasing *Leaf Curling Responsiveness*.	[211]
	*LncRNA42705*, *LncRNA08711*	up	Tomato	*Phytophthora infestans*	*MYB*	Enhance tomato resistance to disease by decoying miR159 and increasing *MYB* gene level.	[150]
	*Sl-lncRNA15492*	up	Tomato	*Phytophthora infestans*	?	Suppresses Sl-miR482a expression, increases *Sl-NBS-LRR1* accumulation and enhances tomato immunity.	[123]
	*StLNC0004*	up	Potato	*Phytophthora infestans*	*EXT*	Enhances the potato defense by up-regulating the transcription level of *EXT* gene.	[212]
Nematode	*MSTRG1206*.*1*, *MSTRG1600*.*1*	up	Soybean	*Heterodera glycines*	?	Potential role in soybean immune response to soybean cyst nematode.	[215]
	*MSTRG*.*16268*.*1*,*MSTRG*.*17157*.*1*	up	Soybean	*Rotylenchulus reniformis*	?	Potential lncRNAs responsive to the *Rotylenchulus reniformis* invasion.	[215]
	*MSTRG*.*2115*, *MSTRG*.*30599*, *MSTRG*.*30601*, *MSTRG*.*31962*	down	Peanut	*Meloidogyne incognita*	?	Probably form network with circRNA320 and MIR482c to enhance the resistance to nematodes.	[216]
	*MSTRG*.*42738*	up	Peanut	*Meloidogyne incognita*	*S1GD6Q*	Probably form network with circRNA226 and *S1GD6Q* to regulate the synthesis of peroxidase and enhance the resistance to nematodes.	[216]
Insect	*LincRNA JAL1*, *LincRNA JAL3*	up	Tobacco	*Manduca sexta*	*WIPK*, *WRKY3*, *WRKY6*, *etc*.	Improve resistance by increasing the accumulation of JAs.	[220]
	*Ya* transcripts	?	*Arabidopsis*	*Myzus persicae*	?	Produced by aphid and translocated into plants to function as lncRNAs to mediate plant virulence.	[223]

ET, ethylene; JA, jasmonic acid; lncRNA, long non-coding RNA; ROS, reactive oxygen species; SA, salicylic acid; TYLCV, tomato yellow leaf curl virus.

### 3.1. Roles of lncRNAs in plant–virus interactions

Viruses are obligate intracellular parasites that seriously threaten plant growth. LncRNAs are involved in the interaction between viruses and their hosts (Fig 3A). This interaction is mutual, with some lncRNAs helping the host to perform antiviral functions, while other lncRNAs are induced by the pathogen or directly encoded by the pathogen and facilitate the replication of virus, weaken the immune system, and even evade immune defenses (Fig 3A). *Tomato yellow leaf curl virus* (TYLCV) causes leaf curl disease in several crops. Identification of lncRNAs in a resistant tomato cultivar following TYLCV infection has highlighted the role of lncRNAs during viral pathogenesis [88]. A total of 1,565 lncRNAs were predicted to be involved in TYLCV infection, among which the lncRNAs *slylnc0049* and *slylnc0761* (which were significantly up-regulated by TYLCV infection) were selected for verification. The accumulation of TYLCV CP increased 200- and 6-fold in *slylnc0049-* and *slylnc0761-*silenced plants [88]. Another study revealed that silencing of *lncRNA0957* resulted in reduced disease severity and viral load of TYLCV in susceptible tomato varieties [185]. In response to *Rice black-streaked dwarf virus* infection, 17 up-regulated and 5 down-regulated lncRNAs were identified. These lncRNAs are probably associated with viral infection probably by regulating the expression of defense-related mRNAs [186]. In *Arabidopsis*, the lncRNA *SABC1* represses *Arabidopsis* immune responses to TuMV, and the accumulation of *SABC1* decreases upon TuMV infection to promote plant resistance [45]. Meanwhile, the lncRNA *AP2*, which negatively correlates with the *APETALA2* (*AP2*) gene, is significantly up-regulated by the infection of *Turnip crinkle virus* and promotes the infection of *Turnip crinkle virus* [187].

RNA silencing plays major roles in plant resistance to viruses [188]. In response to viral infection, some lncRNAs are induced and inhibit the function of miRNAs by acting as their target mimics (Fig 3A). The *slylnc0195*, which is significantly induced by TYLCY inoculation, was shown to dramatically increase the mRNA abundance of the corresponding miR166 targets by competing for the binding of miR166 and attenuated virus accumulation [88]. Meanwhile, *slylnc1077* may act as a decoy of miR399 to regulate plant resistance against TYLCV [88]. Moreover, *lncRNA39026*, which is induced by *P*. *infestans* infection, was shown to decrease the expression level of miR168a, and increase the level of the *SlAGO1* gene [189]. Since AGO proteins play important roles in virus resistance, *lncRNA39026* might play a role in virus resistance. Correspondingly, viruses are able to produce vsiRNAs to silence host lncRNAs to promote viral disease development. The tomato lncRNA *SlLNR1* is targeted by TYLCV-derived siRNA with almost perfect complementary match and silenced, thereby attenuating host antiviral immunity [190]. However, studies on other viruses apart from TYLCV are also very limited and restricted to only transcriptomic studies. *SEAIRa*, an antisense intragenic lncRNA that generated from the 3′ end of SE, represses the expression of SE, a core component of the miRNA biogenesis pathway [171]. However, its roles in plant resistance to viruses and other pathogens have not been determined. Hopefully, more studies on functional characterization of identified lncRNAs will bear interesting results in the future.

Intriguingly, many defective/defective interfering (D/DI) RNAs, satellite RNAs, and even incompletely degraded viral genomic RNAs are considered to be lncRNAs [191–194] (Fig 3A). They have the non-protein-coding features and are involved in the host–virus interactions. For example, citrus tristeza virus (CTV) produces a lncRNA called *low molecular weight tristeza 1* (*LMT1*), which is involved in maintaining the accumulation, movement, and infectivity of the virus by lowering the production of SA and reactive ROS required for antiviral defense [195]. CMV Y- and Q- satRNAs, which are 300 to 400 nt in size and do not encode any functional protein, probably function as lncRNAs [192]. CMV Y-satRNA functions as an siRNA precursor to produce Y-sat siRNAs and targets the host *ChlI* mRNA to bring in bright yellowing symptoms in tobacco, while CMV Q-satRNA can bind to a bromodomain-containing protein (BRP) and probably plays a role in histone remodeling [192,196]. However, the virulence of CMV Y-satRNA results from sRNAs derived from satRNAs, and the role of CMV Q-satRNA has not been verified, which makes it controversial to group these satRNAs as lncRNAs [197,198]. Plant satellites of other viruses, including *Tobacco ring spot virus* satRNA, RNA C, D and F of *Turnip crinkle virus*, and *Cymbidium ring spot virus* satRNA, all possess features of lncRNAs and generate disease symptoms in infected plants, but have not yet been studied as a lncRNA [199–202]. With the study on further discovering the mechanism of satRNAs, there might be more solid evidence to link satRNAs with lncRNAs.

### 3.2. Roles of lncRNAs in plant–fungi interactions

Fungi are eukaryotic pathogens that cause serious diseases to crops. At present, emerging evidence has shown that lncRNAs play important regulatory roles in plant immunity upon the infection of many fungal species (Fig 3B and Table 1). In *Arabidopsis*, 15 lncNATs and 20 lincRNAs were identified to be differentially expressed in response to infection with *Fusarium oxysporum*, a soil-borne plant fungal pathogen, and some of these lncRNAs were demonstrated to affect disease development, probably through their associations with neighboring genes [87]. In wheat, lncRNAs participate in plant immunity in the response to powdery mildew and stripe rust infection [89,203]. Seventy-one wheat lncRNAs were identified in response to powdery mildew infection. These lncRNAs displayed tissue-specific expression patterns, and some of them functioned in plant immunity through their feature as miRNA precursors [89]. In *Brassica napus*, 41 lncRNAs have been identified to respond to *Sclerotinia sclerotiorum* infection, and they probably function as precursors of miRNAs to produce miRNAs such as miR156 and miR169 [54]. Likewise, a further study identified 254 differentially expressed lncRNAs in response to *Blumeria graminis* f. sp. *tritici* stress and 52 lncRNAs in response to *Puccinia striiformis* f. sp. *tritici* in *Triticum aestivum*. Some of these lncRNAs were predicted to be the targets or target mimics of miRNAs and regulate wheat resistance to powdery mildew and stripe rust stress via miRNA regulation [90] (Fig 3B). The roles of lncRNAs in plant antifungal defense networks were also determined in *Vitis vinifera* (grapevine) responses to *Erysiphe necator* (powdery mildew, PM) and *Plasmopara viticola* (downy mildew, DM), and 71 PM- and 83 DM-responsive *V*. *vinifera* lncRNAs were identified [204]. These lncRNAs and their associated protein-coding genes are involved in the modulation of basal and specific defense responses. However, the exact roles of these lncRNAs in plant–fungi interactions and the underlying mechanism are largely unknown. A recent study showed that lncRNAs mediate plant resistance against fungi through their regulation of the JA pathway. *GhlncNAT-ANX2* and *GhlncNAT-RLP7* (Fig 3B) in cotton promote *V*. *dahliae* and *B*. *cinerea* infection, probably by decreasing the expression of their neighboring genes *ANX2 and RLP7*, respectively, exhibiting associations with the decreases in JA pathway genes, *LOX1* and *LOX2* [183], while *GhlncLOX3* positively regulates plant defense against *V*. *dahlia*, exhibiting associations with the increased levels of *GhLOX3 expression* and JA content [173] (Table 1).

### 3.3. Roles of lncRNAs in plant–bacteria interactions

In addition to viruses and fungi, bacteria are another major threat to plants, causing serious yield loss. Studies have demonstrated the involvement of lncRNAs in bacterial disease resistance (Fig 3C and Table 1). Bacterial canker disease of kiwi fruit is caused by the *Pseudomonas syringae pv*. *actinidiae* (*Psa*). The up-regulation of lncRNAs and their interactions with various signaling and defense-related genes have been reported in *Psa*-infected kiwi fruit [205]. A total of 110 lncRNAs responding to *phytoplasma* infection have been identified in *Paulownia* by high-throughput sequencing [206]. When the interaction between tomato and *Ralstonia* s*olanacearum* was studied, 23 differentially expressed lincRNAs were identified. These lncRNAs were found to respond to bacterial wilt infection, probably by their involvement in JA and ethylene signaling pathways, or by regulating the expression of the AGO protein [207]. *Dickeya zeae* responsive lncRNAs were also identified in rice (*Oryza sativa L*.) [208]. Through genomic-wide analysis, 2,518 and 2,191 predicted lncRNAs were found to be up- and down-regulated in response to *D*. *zeae* infection, respectively. Several of these lncRNAs are known to participate in rice immune systems as target mimics of miRNAs. In *Arabidopsis*, 12 lncRNAs react to the infection of *Pst* D3000 [45]. Among them, the *lncRNA SABC1*, which plays negative roles in plant defense by inhibiting the transcription of its neighboring gene *NAC3* and reducing SA biosynthesis, was suppressed in response to *Pst* D3000 infection to activate the plant immunity. The lncRNA *ELENA1* enhances the resistance of *Arabidopsis* to *Pst* DC3000 by interacting with the mediator subunit 19a and FIB2 to promote the gene expression of PR1 [148,149]. The lncRNA *SUNA1*, the expression of which is triggered by SA, also plays a positive role in *Arabidopsis* defense against *Pst* DC3000 [209]. *SUNA1* appears to regulate plant defense by interacting with fibrillarin to enhance the pre-rRNA processing and translational efficiency of some defense genes (Table 1). In addition, the accumulation of large amounts of rice lncRNAs was shown to be significantly altered upon the infection with *Xoo*. The lncRNA *ALEX1* enhances *Oryza sativa* resistance to *Xoo* by up-regulating the endogenous levels of JA and the expression of JA-responsive genes [180].

### 3.4. Roles of lncRNAs in plant–oomycete interactions

Oomycetes are filamentous microbes that represent one of the biggest threats to crops. Among the ubiquitous and highly diverse species of oomycetes, *P*. *infestans* is most notorious, as this oomycete causes late blight of tomato and potato and is blamed for the cause of the Irish potato famine [210]. In tomatoes, more than 600 differentially expressed lncRNAs were identified in response to *P*. *infestans* infection [132]. Tomato *lncRNA16397* and *lncRNA33732* were found to regulate plant defense against *P*. *infestans* by mediating ROS accumulation [132,133], while *lncRNA39026* increased resistance by inducing the expression of PR genes [189] (Fig 3D and Table 1). Furthermore, many lncRNAs have been reported to modulate the defense response to *P*. *infestans* by regulating the function of miRNAs (Fig 3D and Table 1). *LncRNA39026*, *42705*, *08711*, *40787*, *15492*, *23468*, and *08489* positively regulate plant resistance against *P*. *infestans* by acting as competitive endogenous RNAs of miR168a, miR394, miR159, miR482a, miR482b, and miR482e-3p, respectively, while *lncRNA39896* negatively regulates resistance to *P*. *infestans* through its action on miR166b [122–124,184,189,211]. In potatoes (*Solanum tuberosum* L.), 133 differentially expressed lncRNAs were identified in response to *P*. *infestans* infection [212]. Among them, *StLNC0004* suppresses the growth of *P*. *infestans* in *Nicotiana benthamiana*, probably by regulating the transcriptional level of the *EXT* gene.

In addition to *P*. *infestans*, lncRNAs also involve in the resistance to other oomycetes. The differentially expressed pepper lncRNAs in response to *P*. *capsici* were found to increase pepper resistance to soil-borne diseases by interacting with their coordinated miRNA-mRNA and regulating the expression of disease-defense–related genes [213] (Fig 3D and Table 1). Genes encoding zinc finger proteins, pentatricopeptide repeat-containing proteins, and LRR receptor-like serine/threonine-protein kinases are potentially regulated by lncRNAs to regulate plant immune responses to *P*. *capsici* [213]. On the other hand, the expression levels of lncRNAs in oomycetes were also altered during their infection of plant. Eighty-five *P*. *sojae* lncRNAs were found to exhibit different transcriptional patterns 3 h after inoculation onto susceptible soybean leaves compared to their transcription in other growth stages, including mycelia, zoospores, and germinated cysts [214]. A high proportion of these lncRNAs associated with effector-coding genes.

### 3.5. Roles of lncRNAs in plant–nematode interactions

The invasion of nematodes may affect the growth and development of plants, leading to plant deformity. Genome-wide identification and functional deciphering has revealed the involvement of lncRNAs in the responses to nematodes in different plants [215–218] (Fig 3E and Table 1). However, the action mechanisms of these lncRNAs are not clear. The mechanism reported most frequently is that lncRNAs interacts with their corresponding miRNAs and exhibit miRNA-related regulatory effects. For example, in soybeans, 384 and 284 potential lncRNAs were identified in response to 2 nematode species, *Heterodera glycines* and *Rotylenchulus reniformis*, respectively, and 15 and 6 lncRNAs were predicted to be involved in the regulation of nematode-responsive gene expression by their interactions with miRNAs [215]. In response to root-knot nematode stress, 10 peanut lncRNAs were identified to participate in defense-related processes [216]. These lncRNAs formed a regulatory network with corresponding miRNAs and mRNAs, and engaged in peroxidase activity, the lignin biosynthetic process, and oxidation–reduction processes. In the tomato response to *M*. *incognita*, 43 up-regulated and 35 down-regulated lncRNAs were identified, 12 of which were predicted to be sponges of their corresponding miRNAs and to regulate tomato resistance [217]. In rice (*Oryza sativa*), lncRNAs responsive to *Meloidogyne graminicola* infection were predicted to regulate the expression of genes involved in phosphotransferase activity and influence DNA methylation levels in *cis* [219]. These studies revealed the great potential of lncRNAs in plant resistance to nematodes. However, to explore effective plant protection strategies against parasitic nematodes, further studies on specific lncRNAs are needed to confirm the functions of these lncRNAs.

### 3.6. Roles of lncRNAs in plant–insect interactions

Recent studies have also revealed the involvement of lncRNAs in plant resistance to insects (Fig 3F and Table 1). A large number of tobacco lncRNAs were found to be induced by the phytophagous insects *Manduca sexta* [220]. Silencing of the lncRNAs *JAL1* and *JAL3* attenuated plant resistance to *M*. *sexta*, probably through their roles in inhibiting the accumulation of JA and JA derivatives. In addition, a total of 238 armyworm (AW)-responsive lncRNAs were identified in monocot rice, and one lncRNA was the antisense transcript of the JA ZIM-domain gene JAZ10 [221]. A total of 606 differentially expressed lncRNAs were identified in cotton upon the infestation of whitefly [222]. Among them, *lncA07* and *lncD09* potentially increased plant resistance to insect infestation through their regulation of JA content. Intriguingly, during the interaction between plant and insect, RNAs from the insect can translocate into plants and function as virulence factors. *Ya* transcripts from aphid *Myzus persicae* translocate into plants during aphid feeding and migrate systemically to distal leaves in several plant species [223]. *M*. *persicae* that feed on *A*. *thaliana* expressing *Ya1* RNA show increased fecundity. *Ya1* acts as an aphid lncRNA virulence factor to modulate plant processes.

### 3.7. Future perspectives

In summary, compared with the adequate databases and well-developed bioinformatic tools for mRNA, sRNA, and protein, plant-related lncRNA databases are relatively small in number, and their annotation is insufficient, which makes it difficult to study lncRNAs systematically. Moreover, the low abundance, high diversity, and the specific function of each lncRNA also increase the difficulty of discovering the functions of lncRNAs and exploring the underlying mechanisms. Therefore, research on the roles and actions of plant lncRNAs in plant immune responses and other biological processes is at a relatively early stage.

On the other hand, genome-wide analysis has identified plant lncRNAs that are induced or repressed upon stress. Emerging studies on lncRNAs have revealed the essential roles of lncRNAs not only in cellular and developmental processes but also in stress responses, hormone signaling, and pathogenesis. LncRNAs have unique characteristics that make them important players in plant immunity responses [5]. The non-protein-coding nature of lncRNAs allows them to evolve more rapidly than protein-coding genes, and this rapid evolution can lead to the emergence of new lncRNAs with specific functions in plant–pathogen/insect arms races. Moreover, lncRNAs can react to stress responses more rapidly than protein-coding genes, which can be important in the early stages of immune responses when rapid action is needed. By linking early and later immune responses, lncRNAs can play a key role in shaping the overall immune response of the plant. The highly cell type-specific expression of lncRNAs can also regulate the expression of immune and growth genes in different cells, providing an elegant balance between growth and immunity as some protein-coding genes [224]. Overall, the unique characteristics of lncRNAs make them important players in plant immunity responses, and studying their functions can provide insights into the complex interplay between growth and immunity in plants.

As stress responses manifest the accumulation and actions of lncRNAs, more studies should focus on the roles of lncRNAs in plant immune responses. In addition to the genome-wide analysis of the accumulation alternations in lncRNAs upon the infection/infestation by different pathogens/insects, we need to pay more attention on the detailed actions and roles of lncRNAs in immune responses. As it is still difficult to determine the targets of *trans*-acting lncRNAs, future studies may focus on *cis*-acting lncRNAs in plant immune responses, especially on lncRNAs generated from loci close to key immune response genes. With an increasing number of reports on the functional characterization of plant lncRNAs, we may be able to draw an lncRNA regulatory network with protein-coding genes in plant immune responses. At that time, we will be able to identify the differences and correlations between lncRNAs and protein-coding genes in plant immune responses. In addition to the unique roles of hormones, Ca^2+^, protein-coding receptors, protein-coding transcription factors, and other well-defined biomolecules, lncRNAs may perform unique roles in plant immune systems. Studies on lncRNAs may uncover many mysterious phenomena and improve our understanding of plant immune systems.
